# Managing Autism Spectrum Disorder in the Face of Pandemic Using Internet-Based Parent-Mediated Interventions: A Systematic Review of Randomized Controlled Trials

**DOI:** 10.3390/children9101483

**Published:** 2022-09-28

**Authors:** Iyus Yosep, Stephanie Amabella Prayogo, Kelvin Kohar, Hubert Andrew, Ai Mardhiyah, Shakira Amirah, Sidik Maulana

**Affiliations:** 1 Department of Mental Health Nursing, Faculty of Nursing, Universitas Padjadjaran, Bandung 45363, Indonesia; 2 Faculty of Medicine, Universitas Indonesia, Depok City 16424, Indonesia; 3 Department of Pediatric Nursing, Faculty of Nursing, Universitas Padjadjaran, Bandung 45363, Indonesia; 4 Professional Nursing Program, Faculty of Nursing, Universitas Padjadjaran, Bandung 45363, Indonesia

**Keywords:** autism spectrum disorder, internet-based parent-mediated intervention, COVID-19, systematic review

## Abstract

ASD is a neurodevelopmental disorder that is primarily treated with psychosocial intervention. However, it is costly and requires extensive resources to be effective. This inaccessibility is also further worsened by the ongoing COVID-19 pandemic, making the shift to a digital approach a sensible option. Among the available ASD therapies, parent-mediated interventions (PMIs) have a broad application and lower implementation cost. Hence, this systematic review aims to evaluate the potential that telehealth-based PMI holds and explore its feasibility throughout the COVID-19 pandemic. To build up this study, a systematic search through PubMed, Scopus, ProQuest, Wiley, and Cochrane was performed until 14 January 2021. Using the preferred Reporting Items for Systematic Review and Meta-Analysis guidelines, we ultimately included six studies in the review. Each study was evaluated utilizing the Cochrane Risk of Bias (ROB)-2 tool. Generally, parents’ outcomes (knowledge, satisfaction, and compliance) were higher in intervention group (E-learning) compared to control (standard treatment or wait-list). Children also showed some improvements in social skill, communication skill, and intelligence after receiving the treatment. In addition, coaching or therapist sessions were found to be crucial as adjuvant to support parents during the intervention. In conclusion, internet-based parent-mediated interventions are promising and recommended for managing ASD patients, in the face of pandemic. However, more variety in study locations is also needed, particularly in low- and middle-income countries, to tackle the knowledge and clinical application gap. Further research should be conducted with a uniform measurement tool to achieve the same perception and reliable pooled analysis.

## 1. Introduction

Autism Spectrum Disorder also abbreviated as “ASD” refers to a neurodevelopmental condition that is characterized by difficulties in restricted interests, social communication and interactions, and repetitive behaviors [1]. Most ASD children have impaired adaptive skills, while others exhibit psychiatric comorbidities or symptoms. As such, ASD poses a significant threat to patients’ and their families’ quality of life (QoL) [2].

While pharmacotherapy for ASD exists, a set of side effects accompanies its use. Intensive psychosocial intervention is still considered as the gold standard treatment in the management of behavioral symptoms in patients with ASD [3,4]. In children, such intervention at an early stage can improve behaviors, such as shared attention, language, and social interaction, which may potentially impact development and, in turn, lessen the severity of symptoms [1,5].

However, psychosocial interventions are not without their downsides. The therapies are costly and require extensive resources to be implemented effectively. While ASD rates in urban and rural areas are similar, ASD services in the latter are lacking. Geographic distance and low socioeconomic status have been cited to deter families from seeking appropriate services [6].

Considering the ongoing COVID-19 outbreak, access to ASD therapy is further hindered. Many children and their families experience disruption to their ASD therapy [7]. The willingness of health workers to shift to a digital or online approach to ASD behavioral therapy may open new opportunities in the management of ASD and fulfill the need of the hour (or even be implemented henceforward) [5,8].

Of the available ASD therapies, parent-mediated interventions (PMIs) have garnered interest over the past decades. In this type, the parents implement interventions in a tailored manner, enabling them to work on specific skills or certain impairments. As parents spend much time with their children throughout the day, the advantages of PMI include its lower cost and broad application [9].

Consequently, this systematic review was conducted to evaluate the potential of telehealth-based PMI and explore its feasibility throughout the COVID-19 pandemic.

## 2. Methodology

### 2.1. Design Study

The guidelines for the Preferred Reporting Items for Systematic Review and Meta-Analysis were utilized in the process of carrying out the systematic review [10]. This study was registered in the PROSPERO with ID registration number is CRD42022360710.

### 2.2. Search Strategy

A literature search was carried out by the individual through a number of databases, including PubMed, Scopus, ProQuest, Wiley, and Cochrane. Any inconsistencies that were found were brought up for further discussion among the authors. In each database, the search terms used were PubMed: (internet OR web OR telehealth OR mobile application OR virtual OR video OR psychoeducation) AND (“Autism Spectrum Disorder”[Mesh] OR ASD) AND “Parents”[Mesh]; Scopus: Ti-Abs-Key (internet OR web OR telehealth OR mobile application OR virtual OR video OR psychoeducation) AND (“Autism Spectrum Disorder” OR ASD) AND “Parents”; ProQuest, Wiley, and Cochrane: (internet OR web OR telehealth OR mobile application OR virtual OR video OR psychoeducation) AND (“Autism Spectrum Disorder” OR ASD) AND “Parents”. Subsequently, duplicates were removed, and the results were screened using the pre-determined eligibility criteria.

### 2.3. Study Eligibility Criteria

Using the eligibility, inclusion, and exclusion criteria, during the screening process, all of the articles that were found were sorted. The inclusion criteria followed the PICO Framework (Population, Intervention, Comparison, Outcome, and Study), namely:

(1) Population: parents with ASD children.

(2) Intervention: internet-based parent-mediated intervention.

(3) Comparison: usual care.

(4) Outcomes: parent and children’s outcomes related to managing ASD.

(5) Study: randomized controlled trials.

Moreover, studies were excluded if they fulfilled any of the following criteria: (1) animal studies, (2) inaccessible full-text articles, and (3) non-English articles.

### 2.4. Study Selection

All of the articles were looked at by different authors using the PRISMA guidelines. The screening process began with the title, moved on to the abstract, and then went on to the full text of some of the studies to get rid of the ones that did not meet any of the exclusion criteria. In the end, validation was performed on all of the studies that had been chosen to ensure their qualification for the following step.

### 2.5. Data Extraction and Quality Assessment

The authors all collected the data that was available from the studies that were included. The following information, which was taken from each of the studies, the investigators obtained the rated outcomes data, which are divided into two categories: patients’ outcomes and parents’ outcomes. Parents’ outcomes are knowledge, satisfaction, and compliance. Meanwhile, children’s outcomes are social skill, communication skill, and intelligence.

The Risk of Bias (Rob 2) tool by Cochrane was used to evaluate each study’s methodological quality. This tool looked at five performance characteristics: randomized processes, deviations from intended interventions, missing outcome measures, standard measure of outcome, and selection of results reported [11].

## 3. Results

### 3.1. Study Selection

**Figure 1:**
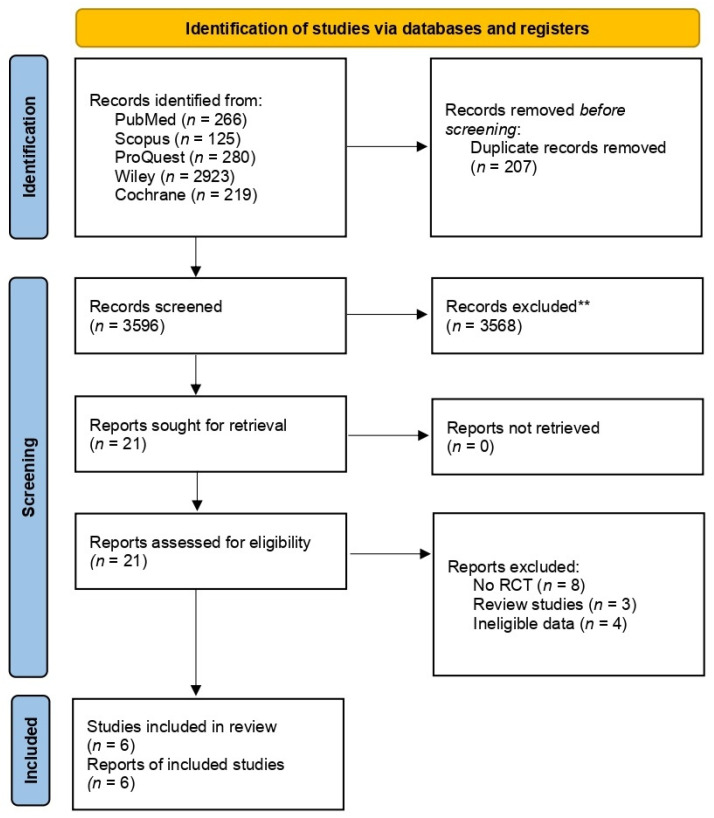
PRISMA flow diagram.

A total of 3813 records were yielded after the preliminary investigation. After deduplicating 207 articles, the authors screened the titles and abstracts, and 21 articles were obtained to be assessed afterwards at full-text level. In addition, we excluded 15 studies due to study design mismatch, namely the studies not being RCT (*n* = 8), review studies (*n* = 3), and ineligible data (*n* = 4). Thus, six articles were ultimately included in this study (Figure 1).

### 3.2. Characteristics of Included Studies

The included studies were all randomized controlled trials (RCT) which were published between 2016 and 2021. Out of six studies, four were conducted in the United States [12,13,14,15], one in Brazil [16], and one study did not mention the study location [17]. A total of 324 participants, who are parents with a child diagnosed with ASD, were randomly assigned to the intervention group and the control group. Most intervention groups were given telehealth or internet-based training in the form of modules, consisting of instructional videos, quizzes, exercises, and others. The intervention was also frequently supported by coaching sessions by a trained therapist via online application. Meanwhile, in the control group, participants received treatment as community standard. In three studies, the control group still received E-learning modules, but differ from its intervention by access duration, delay, and coaching sessions [10,12,15]; the details of the study characteristics are presented in Table 1.

### 3.3. Quality Assessment of Included Studies

**Figure 2:**
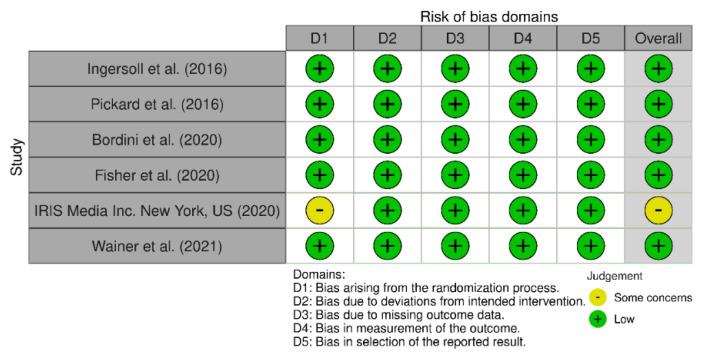
Risk of bias assessment [12,13,14,15,16,17].

To assess the quality, the authors used the Cochrane Risk of Bias. In total, five out of six studies reported low risk of bias, and only one study indicated some concerns of bias (Figure 2).

### 3.4. Parents’ Outcomes

The three aspects of parents’ outcomes assessed in this study were knowledge, satisfaction, and compliance. In the post-test, all participants in the E-learning group achieved higher knowledge score on applied behavior analysis and acceptance commitment training measured, self-efficacy, and the Early Intervention Parenting Self Efficacy Scale (EIPSES) tool. The same result mostly happened in satisfaction and compliance aspects; the participants would even recommend the modules to others.

Parent’s knowledge about autism is crucial for early diagnosis and appropriate treatment of ASD. To analyze this aspect, a study by Wainer et al. used EIPSES tools, which consisted of a 20-item questionnaire including parent competence and their outcomes expectations. Parents who participated in training conducted online acquired a greater number of parenting skills than those who participated in standard community treatment. Therefore, this correlates significantly with higher EIPSES outcome [15]. Another study by Ingersoll et al. showed that both the self-directed teaching and therapist-assisted group obtained higher self-efficacy in the post-test (61.43 ± 13.27 vs. 54.69 ± 14.81 and 58.62 ± 12.12 vs. 53.23 ± 13.14, respectively). However, therapist-assisted groups gained more advantages, such as positive perceptions and providing feedback on their intervention [17].

### 3.5. Children’s Outcomes

There were four aspects related to children’s outcome that were assessed in this study, namely social skill, communication skill, intelligence, and autism symptomatology. The result of children social skill measurement showed variety among studies [13,16,17,18]. Two studies reported negative and inferior effects from intervention, while the remaining showed the opposite. All ASD children showed improvement in communication skill and intelligence after receiving intervention compared to control. In addition, studies also found significant increase in children intelligence related with IQ, work skills, play skills, vocabulary, daily living skills, as well as motor skills [18].

Children’s outcome related to the improvement of communication skill, social skill, and intelligence are shown in this study. Bordini et al., the only study included which measured IQ improvement, showed significant increase in non-verbal IQ compared to standard community treatment [16]. This result is also supported by Fisher et al. and Ingersol et al., who saw improvements in work activities, play activities, as well as vocabulary skills [13,17]. The details of the study outcome are presented in Table 2. 

## 4. Discussion

Our study found that PMIs have the potential to increase parents’ knowledge, satisfaction, and compliance. ASD children who received the internet-based parent-mediated intervention also improved social skills, communication skills, and intelligence after receiving the treatment.

A similar systematic review by Nocker et al. using telehealth for children with ASD reported significant positive outcomes for children [19]. Improving ASD children’s outcome is a challenge, especially in IQ measurement. Patients who have a lower IQ than average generally have a poorer response to intervention. This was shown to be the case in a clinical trial that was conducted by Scahill et al. that examined 24 individuals with face-to-face sessions. The trial concluded that children with ASD required more intensive and longer interventions [20]. A systematic review by Pacia et al. assessing social communication interventions for children with autism showed that most studies reported successful generalization of skills, which indicated that telehealth-related interventions are promising in clinical practice [21]. Using Vineland Adaptive Behavior Scales (VABS-I) and others, our included study found significantly higher score in intervention group compared to standard community treatment. However, compared to the self-directed group, the result was not significantly higher. The efficacy of telehealth was compared to face-to-face intervention and showed no significant difference in communication and social skills [19]. Regarding customer satisfaction and compliance, participants were satisfied overall with E-learning, be it as a self-directed or therapist-assisted approach. Another similar systematic review involving 14 studies by Guaiana et al. assessed the use of telepsychiatry in depression. The study showed that satisfaction is equivalent or significantly higher in the telemedicine group compared to face-to-face intervention [22]. This finding indicates telehealth might serve as alternative or adjunct to traditional delivery models in medicine, with a potential to deliver treatment in effective, acceptable, user-friendly, and cost-efficient manner [23]. However, another similar exploratory open study by Bonnot et al. using mobile application for caregivers of ASD children found a disappointing result, that is the application was only intensely used during the first six months. In addition, only 46% of participants maintained completion rates that were greater than 50%, and 54% of users did not use the application on a regular basis [24]. This phenomenon is known as “law of attrition” in eHealth trial, in which a proportion of users choose to drop out before completion [25]. As a solution, Bonnot et al. suggests to regularly remind, motivate, as well as provide feedback to parents via coaching or therapy sessions [24].

PMIs engage parents into a therapist role to implement specific therapies to their own child. Parents will learn therapy techniques from professionals, including teaching comprehensive skills and others, such as communication skills, social skills, or joint attention. Parents who spend a lot of time with their child will create a consistent reinforcement and training, as well as a direct implementation to daily life. This shows an opportunity for a cost-effective intervention [9,26].

In high-income countries, PMIs have been considered as a cost-effective treatment and recommended to be used in children with neurodevelopmental disorders (NDDs), including ASD. On the other hand, low-income countries, such as the ones in South Asia, have less access to professionals and educator specialized for NDDs can use PMIs as a pragmatic strategy to support their child. Furthermore, compared to behavioral approaches and home treatment, PMIs were found to lessen the demands on ASD children. A meta-analysis conducted by Conrad et al. has also proven the benefit in adaptive functioning and disruptive behaviors rated by parents after PMIs compared to no PMI [9,27].

Conventional PMIs were usually conducted in the family home with the therapist. This kind of formal PMIs are rare as there are limitations in community, e.g., lack of professionals, lengthy waitlists, rural area with limited financial and transportations, and time limitations. Adding to this, the COVID-19 pandemic situation has also limited human contact, restricting this traditional way to be sustained. Considering these facts, an adaptation of PMI methods is required [17].

With the advancement of technology, videos or online training are readily available and accessible via the internet. Another kind of therapy, such as cognitive behavioral therapy delivered via telehealth, has given an equally effective therapy [28]. One example of online PMIs is ImPACT, a coaching program for parents with ASD children, which is based on applied behavioral analysis (ABA) principles available online through https://www.project-impact.org (accessed on 3 December 2021). This program can either be completed independently or with the assistance of a therapist through the use of web-based remote training. Through this program, parents can obtain certification after finishing the beginner and advanced courses, receive consultation from trainer consultant for 6 months while using the program, and meet fidelity of implementation [17,29].

Moreover, the implementation of this internet-based system needs to pay attention to ethical and legal aspects, yet important gaps remain between technological capabilities and legislation/regulations [30]. The current pandemic is the stepping stone for starting an internet-based system. To prevent ethical problems, there needs to be further discussion and implementation regarding informed consent, data protection, confidentiality, and regulation regarding telemedicine. Thus, the fundamental methods and solutions have to ensure data confidentiality, integrity, and availability to follow government regulations that apply in a country. Another important aspect that needs to be considered is the training and expertise of the individuals who carry out internet-based intervention. Long-term and short-term preparations are needed, including responsibilities in training to establish Internet-based interventions as quickly as possible [30,31,32].

Our study proposes telehealth supported by therapist or coaching sessions as an alternative treatment. However, the authors admit there are some limitations in this systematic review and the included studies, such as small sample size and undistributed study location (in America). Therefore, its clinical applications in other parts of the world, especially in rural or urban areas are still unknown. In addition, it is also impossible to perform pooled data analysis due to the variation of data reported. Furthermore, the heterogeneity of the study may be substantial, indicating the need for the same study and study protocol. Despite the limitations stated above, we only included randomized clinical trials, which are the best study design for interventional studies.

## 5. Conclusions

In conclusion, internet-based parent-mediated interventions are relatively effective as shown by the better results in parents’ knowledge, satisfaction, and compliance as well as children’s social skill, communication skill, intelligence, and autism symptomatology. Thus, they are recommended for the management of ASD patients considering the current pandemic situation.

Further implementation requires attention in several respects, such as developer assistance in the creation and integration of user-friendly systems to assist the intervention process. Doctors as well as health workers also need to develop protocols that help patients, including parents, meet their children’s needs regarding intervention by taking into account laws and ethics. In the future, the government will be required to make special regulations that support the use of interned-based intervention in patients with ASD to support interventions. However, more distributed studies in other parts of the world and further research are warranted, particularly in low- and middle-income countries, to tackle the knowledge and clinical application gap. Furthermore, a uniform measurement tool is needed to achieve the same perception, as well as providing data for pooled analysis, which is more reliable.

## Figures and Tables

**Table 1:** Characteristics of the included studies.

No.	Authors	Year	Location	Sample Size	Intervention Group	Control Group
1.	Ingersoll et al. [17]	2016	Not mentioned	28 families having a child with ASD	Six-month access to the ImPACT Online website, with 2 coaching sessions per week (30 min each) for 12 weeks from a trained therapist via video conference	Six-month access to the ImPACT Online website
2.	Pickard et al. [12]	2016	United States	28 parents of a child with ASD	Access to 12-lesson intervention program from ImPACT Online website and 2 coaching sessions per week (30 min each (from a trained therapist via video conference (Skype)	Access to 12-lesson intervention program from ImPACT Online website
3.	Bordini et al. [16]	2020	Brazil	67 patients (34 intervention vs. 33 control)	Over 22 sessions of parent training	Standard community treatment
4.	Fisher et al. [13]	2020	United States	25 participants (13 intervention and 12 control)	Virtual private network-based e-learning modules and scripted role play	Waitlist group
5.	IRIS Media Inc. [14]	2020	United States	156 parents of children with ASD	4 modules (7 videos ranging in length from 3 to 13 min) with more explicit information on function-based strategies	Self-directed teaching routine (8 modules with each video ranging 3–5 min in duration)
6.	Wainer et al. [15] (Grey Literature)	2021	United State	20 families	Online Reciprocal Imitation Training (RIT) with four sequential learning modules. Each module includes instructional videos, quizzes, interactive exercises, at home planning, and reflection	Treatment as usual (TAU)

**Table 2:** Study outcome.

No.	Authors	Parents’ Outcomes			Patients’ Outcomes		
		Knowledge	Satisfaction	Compliance	Social Skill	Communication Skill	Intelligence
1.	Ingersoll et al. [17]	Parent self-efficacy post-test was non-significantly higher in therapist-assisted group compared to control (61.43 ± 13.27 vs. 58.62 ± 12.12)	Post-treatment, the therapist-assisted group had significantly more favorable perceptions than the control group (1.60 ± 0.5 vs. 2.06 ± 0.56; *p* = 0.03)	Participant in therapist-assisted group had higher fidelity significantly compared to control (2.52 ± 0.78 vs. 3.39 ± 0.76; *p* < 0.01)	Post-Child social skill with Vineland Adaptive Behavior Scales (VABS-II) in therapist-assisted group was non-significantly higher compared to control (75.71 ± 9.07 vs. 75.33 ± 12.40)	Post-Child communication skill with Vineland Adaptive Behavior Scales (VABS-II) in therapist-assisted group was non-significantly higher compared to control (77.36 ± 13.79 vs. 75.33 ± 12.40)	Post-Child vocabulary was higher in therapist-assisted group compared to control (243.64 ± 237.94 vs. 210.38 ± 187.46); As well as child daily living skills (77.00 ± 11.14 vs. 74.23 ± 10.42) and motor skills (83.14 ± 11.27 vs. 82.85 ± 9.74)
2.	Pickard et al. [12]	-	The intervention content was more well-received by parents in the TA group (6.83 ± 0.25 vs. 6.35 ± 0.73; *p* = 0.03)	Parents in both intervention groups used ImPACT Online similarly (6.49 ± 0.33 vs. 6.22 ± 0.73; *p* = 0.21)	-	TA parents saw more improved performance in their child’s social skills during the program (5.85 ± 0.87 vs. 4.92 ± 0.40; *p* = 0.05)	-
3.	Bordini et al. [16]	-	-	-	Non-significant decrease in Vineland social skill standard score (VABS-I) (58.89 ± 13.37 vs. 56.04 ± 11.09; Effect = −1.16; *p* = 0.685)	Significant increase in Vineland communication standard score (VABS-I) (47.48 ± 7.78 vs. 45.86 ± 9.41; Effect = 2.42; *p* = 0.003)	Significant increase in non-verbal IQ (60.21 ± 9.08 vs. 66.65 ± 19.47; Effect = 7.49; *p* < 0.001)
4.	Fisher et al. [13]	-	On a 7-point scale, mean training satisfaction was 6.6 ± 0.7 (5–7); Mean satisfaction rating with technology used was 6.0 (5.2–6.5); Mean satisfaction rating with the content was 6.6 (6.3–6.5); High satisfaction with their interaction with the researcher (6.7); Participants even indicated they would recommend to others	-	-	-	Significant increase in percentage of skills mastered on the Behavioral Implementation of Skills for Work Activities/BISWA (93% ± 12% vs. 15% ± 9%) and Behavioral Implementation of Skills for Play Activities/BISPA (80% ± 29% vs. 3% ± 10%) compared to the baseline, also compared to the control group in BISWA (35% ± 19%; *p* < 0.005) and BISPA (13% ± 9%; *p* < 0.005)
5.	IRIS Media Inc [14]	Mean Value of Knowledge About Applied Behavior Analysis and Acceptance Commitment Training Measured by Questionnaire was higher in intervention group (14.52 ± 2.7 vs. 13.37 ± 2.4; *p* = 0.007)	Consumer Satisfaction Questionnaire was higher in intervention group compared to control (5.50 ± 0.67 vs. 5.24 ± 0.88; *p* = 0.045)	Mean Value Family Quality of Life Survey was higher in intervention group (3.99 ± 0.5 vs. 3.86 ± 0.6; *p* = 0.81)	Mean Value Scales of Independent Behavior-Revised (SIB-R) after three weeks was higher in intervention compared to control (65.56 ± 15.4 vs. 63.87 ± 16.2; *p* = 0.246). Mean Value Child Behavior Measured by Strengths and Difficulties Questionnaire was non-significantly higher in control group (21.00 ± 5.2 vs. 19.27 ± 5.2; *p* = 0.598)	-	-
6.	Wainer et al. [15] (Grey Literature)	Early Intervention Parenting Self Efficacy Scale (EIPSES) was slightly higher in intervention group (118.19 ± 2.88 vs. 108.33 ± 2.70; *p* = 0.029)	Participants rated the intervention as very safe, effective, user-friendly, good fit to their child and family	There was significant difference in parents’ RIT fidelity compared to control group (4.33 ± 0.27 vs. 1.77 ± 0.26; *p* < 0.001). Family’s quality of life in intervention group was also higher (108.02 ± 2.72 vs. 103.20 ± 2.55; *p* = 0.220)	Unstructured imitation assessment was used to measure child social imitation. The UIA score was higher in intervention group (8.54 ± 1.33 vs. 4.40 ± 1.24)	Social communication checklist (SCC) was significantly higher in children receiving intervention group (146.41 ± 5.72 vs. 129.34 ± 5.35; *p* = 0.048)	-
